# Selective effect of static stretching, concentric contractions, and a balance task on ankle force sense

**DOI:** 10.1371/journal.pone.0210881

**Published:** 2019-01-17

**Authors:** Darjan Smajla, Amador García-Ramos, Katja Tomažin, Vojko Strojnik

**Affiliations:** 1 Faculty of Sport, University of Ljubljana, Ljubljana, Slovenia; 2 Department of Physical Education and Sport, Faculty of Sport Sciences, University of Granada, Granada, Spain; 3 Department of Sports Sciences and Physical Conditioning, Faculty of Education, CIEDE, Catholic University of Most Holy Concepción, Concepción, Chile; University of L'Aquila, ITALY

## Abstract

Proper ankle motor control is critical for balance in the human body during functional activities such as standing, walking, and running. Different exercise modalities are often performed during the same training session where earlier activities may influence later ones. The purpose of the current study was to determine the acute effects of different exercise modalities on ankle force sense. Seventeen subjects performed four different intervention protocols (static stretching, balance task, concentric contractions, and control) in random order. Each session comprised measurements before and after the intervention protocol of the force sense of the ankle plantar flexors (PF) and dorsal flexors (DF) at 10% and 30% of maximal voluntary isometric contraction (MVC). Absolute errors (AE) were calculated separately for each force level and muscle group. An overall PF error (PF-SUM = PF at 10%MVC + PF at 30%MVC), DF error (DF-SUM = DF at 10%MVC + DF at 30%MVC) and ankle error (PF-DF-SUM = PF-SUM + DF-SUM) were also calculated. The main effect of time generally revealed that ankle force sense was significantly reduced after static stretching (PF-DF-SUM: Pre: 6.11±2.17 Nm, Post: 8.03±3.28 Nm; *p* < 0.05), but no significant differences were observed for the concentric contractions (PF-DF-SUM: Pre: 6.01±1.97 Nm, Post: 6.50±2.28 Nm) and the balance task (PF-DF-SUM: Pre: 5.25±1.97 Nm, Post: 5.50±1.26 Nm). The only significant interaction was observed for the PF-DF-SUM (F = 4.48, *p* = 0.008) due to greater error scores after stretching (+31.4%) compared to the concentric (+8.2%), balance (+4.8%), and control (-3.5%) conditions. Based on these results, static stretching should not be performed before activities that require a high ankle force sense such as balance, coordination, and precision tasks.

## Introduction

Awareness of the acute effects of different exercise modalities is important to prescribe them in an optional sequence during a training session. For instance, it is well known that static stretching impairs subsequent physical performance [1]. One the other hand, there is not much information on the acute effects of different exercise modalities (e.g., stretching, concentric contractions, or balance task) on kinaesthesia. Kinaesthesia has been proposed as an important factor in automatic control of movement, balance, and joint stability [2]. Over the past few years, developing kinaesthetic sense has become a necessary element in re-establishing proper motor control after injury [3] and in preventing falls in the elderly [4]. Improvements in kinaesthetic sense within a sports context usually occurs in parallel with other training interventions [5], however, its development and use within an exercise unit have had little attention.

Kinaesthesia refers not only to the sensation of limb position and movement, but also the perception of force produced by the muscles [6,7]. The ability to sense muscle force might be equally important to joint position sense with regards to joint stability [8]. It is well known that force control of submaximal contractions play a crucial role in performing day-to-day activities [9]. The ankle joint plays a significant role in postural stability. Since the ankle is positioned close to the body’s base of support, proper motor control of the ankle is critical for the balance during functional activities such as standing, walking, and running [10]. It has previously been shown that ankle strategies are more important in an anterior-posterior direction in which plantar-flexor (PF) and dorsi-flexor (DF) muscles play an important role [11]. It has been already determined that force sense of the ankle is impaired in people with functional ankle instability [8]. However, little is known about the acute influence of performing different exercise modalities on ankle force sense. There have been some inconsistent results between different studies that could be due to variations between subjects, measuring devices, and intervention protocols [12–15]. In this regard, the influence of exercise on joint kinaesthesia should be further examined.

Different exercise modalities may have different acute influences on the functional state of the muscles, leading to divergent influence on muscle mechanoreceptors and consequently on kinaesthesia and motor control. Static stretching, fatiguing concentric contractions, and different balance tasks are among the most common exercise modalities. To date, investigations on kinaesthesia have mainly focussed on the effects of fatigue. Some studies reported disturbed kinaesthesia [12,16,17] while others suggested that muscle fatigue has no effect [13,18]. Impairment in kinaesthesia could be caused by the accumulation of metabolites in the muscle that can affect muscle spindle activity [19], as well as by changes in functioning of mechanoreceptors [20], and central nervous system [21]. Varying results can also be found for stretching. Acute decreases in kinaesthetic acuity were observed following proprioceptive neuromuscular facilitation stretching (PNF) [14], while others demonstrated that static muscle stretching has no effect on kinaesthetic senses [15,22,23]. Static stretching could alter kinaesthesia because of reduced passive muscle-tendon unit (MTU) stiffness, increased MTU length [24] and changes in neural level (reduced tonic reflex activity) [25]. It is also known that balance training induces spinal and supraspinal adaptations in all sensory systems [26] and evokes sensory reorganization [27], but there is no evidence on the acute influence of balance training on different tests for measuring kinaesthesia. Because of these conflicting results, more research is needed to clarify how the aforementioned exercise modalities influence ankle force sense.

To address these research gaps, the aim of the present study was to assess the reliability of an ankle force sense test and to explore the acute effects of different exercise modalities on ankle force sense. Specifically, these modalities included static stretching, fatiguing concentric contractions, and balance task. We hypothesised that static stretching and fatiguing concentric contractions would have significant impact on the force sense of the ankle.

## Materials and methods

### Subjects

Seventeen students of the Faculty of Sports (8 female, 9 male) (mean ± standard deviation: age = 23.5 ± 1.9 years; body height = 1.74 ± 0.07 m; body mass: 67.6 ± 11.6 kg) volunteered for this study. They had no history of ankle injury or neuromuscular deficits that could compromise the performance being tested. Subjects were excluded if there was history of injury, presence of central nervous system dysfunction, or any acute symptoms of lower extremity pathology. All subjects were physically active, but none of them was a competitive athlete. They refrain from physical activities and alcohol at least 48 hours prior to testing. Subjects were not allowed to consume caffeine on the day of measurement. They were informed about testing procedures and provided a written informed consent prior to commencing the study. The experiment was approved by the local Ethics Committee of the Faculty of Sports Ljubljana according to the Declaration of Helsinki.

### Experimental design

**Fig 1:**
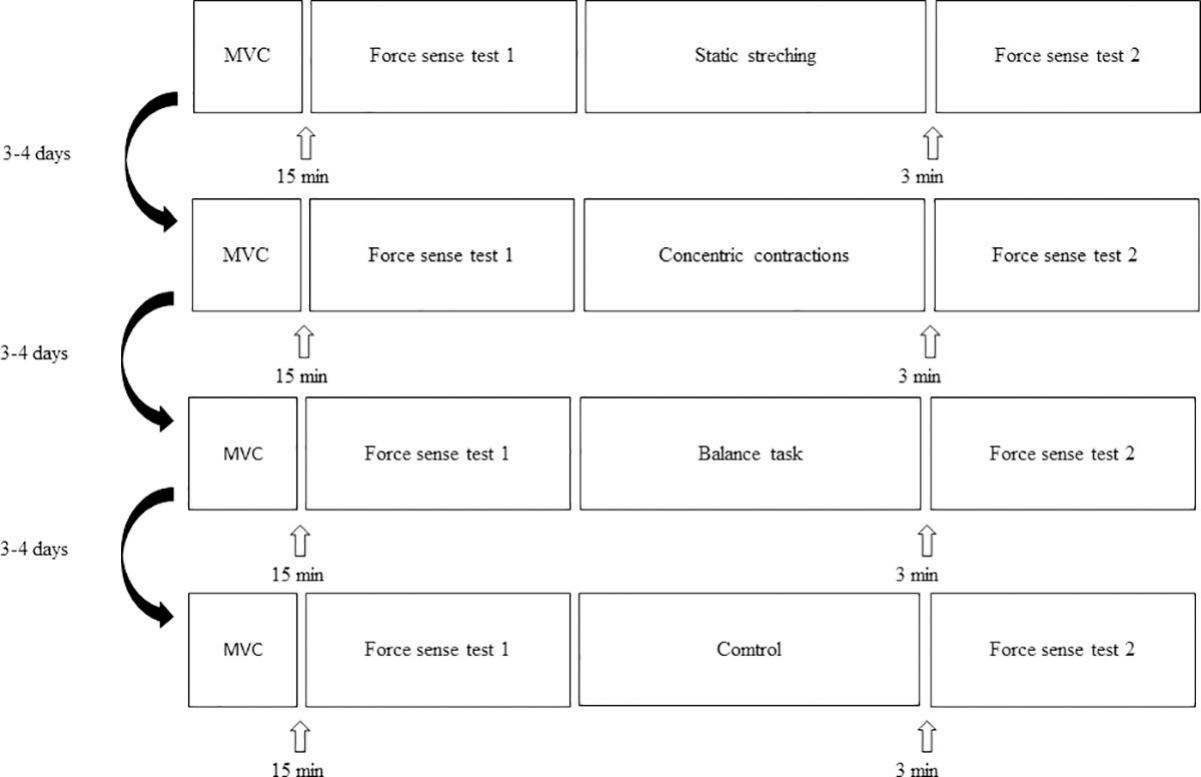
Overview of four experimental sessions. Four exercise modalities were applied in random order.

The study was designed to evaluate the acute influence of different exercise modalities (static stretching, balance task, concentric contractions, control) on ankle force sense (Fig 1). Actual measured values represent torque (Nm), however, because of constant axis and established terminology in the field of kinaesthesia, the term force sense will be used in the article. Subjects attended the laboratory five times, each separated by three to four days. The first session was used for familiarization with the tests and exercise modalities. The acute effects of single exercise modalities were evaluated over the remaining four sessions. The maximal voluntary isometric contraction (MVC) for PF and DF were performed after a warm-up (four graded submaximal contractions). A 15-minute period of rest was given to avoid potentiation. The initial ankle force sense test was then performed, after which the subject performed the exercise modality for that session, with the second force sense test being repeated 3 min later. Before test subjects actively moved ankle through full range of motion to avoid thixotropy [28]. Only the ankle of the dominant leg was analysed. Leg dominance was determined with question: “With which foot would you kick a ball to hit a target?” [29].

### Testing procedures

#### Maximal voluntary isometric contraction (MVC)

A custom designed isometric chair equipped with force sensor (MES, Maribor, Slovenia) was used to test maximal PF and DF isometric strength. Subjects were seated in a chair with hips and knees flexed to 90°. The rotational axis of the force sensor was aligned to the rotational axis of the ankle (i.e. medial ankle malleolus). The leg was held to the isometric chair at the thighbone and around the forefoot (aligned with the head of the fifth metatarsal). The fixed position minimised the involvement of other muscle groups. Subjects were verbally instructed to perform isometric contraction for 5 s with maximum effort. Two trials were performed for PF and another two for DF, with a 30 s rest between trials. The maximal force of the two trials for PF and DF was used for further analysis.

#### Force-sense test

Force-matching procedures were performed unilaterally at 10% and 30% of the MVC previously calculated. Each subject was instructed to develop a determined level of force, first with feedback information and then without feedback. Subject positioning was identical to MVC testing. During the trial with feedback, subjects were instructed to obtain the target force utilizing a computerized digital readout (LabChart 8, ADInstruments, Bella Vista, Australia). They were asked to maintain the contraction for 5 s as near as possible to target force and then relax. Immediately after relaxation, their goal was to reproduce the target force with the same ankle without feedback from the digital readout. Subjects pressed a hand switch when they perceived that the pre-defined target force was reached. Subjects were not able to see their feet during the test. Three trials, consisting of one repetition with feedback and another repetition without feedback, were performed for each force (10% and 30% of MVC) and muscle group (PF and DF). There was a 30 s rest period between each trial. Subjects were not given feedback about their force matching performance. The final second of the target force, and the initial second of reproduction force were used in data analysis, this format having previously been shown as a reliable method of data processing [8]. Absolute error (AE) was used to assess ankle force-matching performance, presenting a measure of the overall accuracy of the ankle force matching performance [12]. The variable was calculated as the difference between the force developed with feedback from the digital readout and the force developed without visual feedback. For statistical analysis we used the average value of the 3 trials for each force (10% MVC, 30% MVC) and PF and DF separately. AE were calculated for PF at 10% MVC (PF 10%), PF at 30% MVC (PF 30%), DF at 10% MVC (DF 10%), DF at 30% MVC (DF 30%). Overall PF errors (PF_SUM) were calculated as the total of PF at 10% MVC and PF at 30% MVC, while overall DF errors (DF_SUM) represented the total DF at 10% MVC and DF at 30% MVC. The total of PF_SUM and DF_SUM together represented the overall average error in the force sense test (PF-DF_SUM).

#### Exercise modalities

Subjects performed three different intervention protocols and one control session in randomized order. Sessions without intervention protocol were used to determine the reliability of the force sense test. During the control sessions subjects were only allowed to walk in the laboratory. The intervention protocols were designed to target muscles around the ankle joint (PF and DF) of the dominant (measured) leg.

Three different static stretching exercises were performed. Each of them targeted a specific muscle (gastrocnemius, soleus, tibialis anterior). For the gastrocnemius muscle, subjects were instructed to place both forearms against the wall. The control leg was placed on the ground in front of the body and with bent knee. Dominant leg was placed behind the body with the straight knee and the heel flat on the ground. Once the subjects adopted this position, they were instructed to lean forward at the hips and stretch the calf muscles. A similar stretching protocol was used for the soleus muscle with the only difference that the knee of the dominant leg was in a bent position. Tibialis anterior was stretched in a standing position with the knees slightly bent. Subjects used the wall in front of them to support balance. Control foot was placed flat on the ground, while the dominant leg was placed behind the stable foot with the toes touching the ground. Subjects were instructed to push the stretching leg forward to feel the stretch through their shins. Each muscle was stretched 6 times for 40 s, with 20 s rest between each repetition. Subjects were instructed to stretch the muscle to their discomfort zone, but not pain, as acknowledge by the subject [30].

The balance task intervention was undertaken as a one-legged stance on Airex soft mat. The task was performed barefoot, with open eyes and slightly bent knees. Arms were crossed on the chest. Subjects performed four sets, each set had four repetitions of 20 s with 40 s rest between repetitions [26]. There was a 1 min rest between sets to avoid fatigue.

The fatiguing concentric contractions intervention included two different exercises. In the first exercise, subjects were asked to perform dorsal flexion of the ankle with a foot strap cable equipment. In the second exercise, they performed toe-lifts in a press machine. They were asked to follow the beat of a metronome (60 beats/min, 1 s for concentric part, 2 s for eccentric part). Loading for both exercises represented the 30% of the one-repetition maximum (1RM) and they performed 30 repetitions per exercise. The 1 RM was calculated from a test of less than 10 repetitions during the familiarization session by way of the Brzycki equation [31]. Subjects performed one set with the maximal number of repetitions. In addition, subjects were given verbal encouragement to ensure that maximal number of repetitions was achieved.

### Statistical analysis

Descriptive data of the dependent variables have been presented as means and standard deviation. Normal distribution of the data was tested using the Shapiro-Wilk test and the homogeneity of variances with Mauchly's sphericity test. Paired sample t-tests and Cohen's *d* effect size (ES) were used to evaluate the systematic differences between both trials performed in the Control condition. Reliability was assessed through the standard error of measurement (SEM) and interclass correlation coefficient (ICC; model 3.1) [32]. Acceptable reliability was determined as an ICC > 0.70 [33] A two-way repeated-measures ANOVA with Bonferroni *post hoc* corrections (time [pre-exercise and post-exercise] × exercise modality [static stretching, fatiguing concentric contractions, balance, and control]) was applied to the absolute error scores of each dependent variable. Statistical significance was accepted at *P* < 0.05 level (two-tailed) and confidence limits were set at 95%. Reliability assessments were performed by means of a custom spreadsheet [34], while other statistical analyses were performed using the SPSS (IBM SPSS version 25.0, Chicago, IL, USA) software package.

## Results

All data was normally distributed (*p* > 0.05) and the homogeneity of variances was confirmed (*p* > 0.05). The reliability analysis revealed no significant differences between Trials 1 and 2 for all the dependent variables (Table 1), while all ICC values showed an acceptable reliability (ICC ranged from 0.79 to 0.92).

**Table 1:** Reliability values for force sense test variables.

Variable	Trial 1 Mean (SD)	Trial 2 Mean (SD)	*P*	ICC (95%CI)
PF 10% (Nm)	2.27 (1.05)	2.09 (1.04)	0.290	0.81 (0.54, 0.93)
PF 30% (Nm)	2.07 (0.74)	2.12 (0.97)	0.740	0.79 (0.51, 0.92)
DF 10% (Nm)	0.70 (0.38)	0.66 (0.43)	0.352	0.91 (0.77, 0.97)
DF 30% (Nm)	0.89 (0.53)	0.85 (0.66)	0.666	0.85 (0.64, 0.94)
PF-SUM (Nm)	4.34 (1.22)	4.21 (1.37)	0.461	0.86 (0.65, 0.95)
DF-SUM (Nm)	1.59 (0.72)	1.51 (0.88)	0.356	0.92 (0.79, 0.97)
PF-DF-SUM (Nm)	5.93 (1.51)	5.72 (1.66)	0.257	0.90 (0.75, 0.96)

*P*, *p-*value obtained from paired samples t tests; ICC, intraclass correlation coefficient; 95% CI, 95% confidence interval.

The only significant interaction was observed for the PF-DF-SUM (F = 4.48, *p* = 0.008) due to greater error scores after stretching (+31.4%) compared to the concentric (+8.2%), balance (+4.8%), and control (-3.5%) conditions (Table 2). The ANOVA test showed significant main effect of time for DF 10% (F = 11.8, *p* = 0.003), PF-SUM (F = 6.52, *p* = 0.021), and DF-SUM (F = 5.00, *p* = 0.040 due to higher error scores post-exercise, but no significant differences were observed for PF 10% (F = 1.16, *p* = 0.298), PF 30% (F = 1.91, *p* = 0.186), and DF 30% (F = 0.99, *p* = 0.335). Differences among exercise modalities were statistically significant for DF 10% (F = 3.63, *p* = 0.019), and DF-SUM (F = 4.66, *p* = 0.006) due to higher error scores in the stretching condition, but no significant differences were observed for PF 10% (F = 1.86, *p* = 0.148), PF 30% (F = 0.76, *p* = 0.523), DF 30% (F = 2.73, *p* = 0.054), and PF-SUM (F = 2.53, *p* = 0.068).

**Table 2:** Comparison of the error scores for force sense between different exercise modalities.

Variable	Stretching		Concentric		Balance		Control	
	Pre	Post	Pre	Post	Pre	Post	Pre	Post
PF 10% (Nm)	2.10 (1.04)	2.67 (1.88)	2.08 (0.99)	2.18 (1.04)	1.60 (0.94)	1.78 (1.01)	2.27 (1.05)	2.09 (1.04)
PF 30% (Nm)	1.98 (0.91)	2.86 (1.84)	2.24 (1.65)	2.30 (1.33)	1.97 (1.05)	1.89 (0.94)	2.07 (0.74)	2.12 (0.97)
DF 10% (Nm)	0.81 (0.52)	1.17 (0.63)	0.83 (0.42)	0.97 (0.50)	0.85 (0.43)	0.94 (0.45)	0.70 (0.38)	0.66 (0.43)
DF 30% (Nm)	1.22 (0.57)	1.34 (0.94)	0.87 (0.53)	1.05 (0.61)	0.84 (0.48)	0.88 (0.57)	0.89 (0.53)	0.85 (0.66)
PF-SUM (Nm)	4.08 (1.60)	5.53 (2.78)	4.32 (1.80)	4.48 (1.92)	3.55 (1.65)	3.67 (0.99)	4.34 (1.22)	4.21 (1.37)
DF-SUM (Nm)	2.03 (0.98)	2.50 (1.19)	1.69 (0.73)	2.02 (0.87)	1.69 (0.66)	1.82 (0.84)	1.59 (0.72)	1.51 (0.88)
PF-DF-SUM (Nm)	6.11 (2.17)	8.03 (3.28)*^,a,b^	6.01 (1.97)	6.50 (2.28)	5.25 (1.97)	5.50 (1.26)	5.93 (1.51)	5.72 (1.66)

Mean (standard deviation). Pairwise comparisons were only performed for PF-DF_SUM because it was the only variable that showed a significant time × exercise modality interaction (*p* = 0.008).

*, significant differences compared to pre-intervention

^a^, significant differences compared to balance

^b^, significant differences compared to control. No significant differences at pre-intervention were observed between the four exercise modalities.

## Discussion

The present study examined the acute effects of static stretching, fatiguing concentric contractions, and balance task on force sense of the ankle. The main findings revealed that: (1) overall AE of the ankle (PF-DF-SUM) was significantly greater after static stretching compared to other conditions, (2) overall AE of DF (DF-SUM) was significantly greater after static stretching compared to control and, (3) AE of DF at 10% MVC (DF 10%) was significantly greater after static stretching compared to control and balance condition. Our results also suggested deterioration of force sense of the ankle after concentric contractions for DF-SUM, but failed to show differences in respect to control condition.

We assumed that changes to the functional state of the muscle following different exercise modalities would influence ankle force sense acuity. Our findings indicate that only static stretching had a significant effect on the ankle, leading to greater AE in the force sense test after intervention. No studies were found presenting direct influence of static stretching on ankle force sense. However, our results do relate to findings from different authors on the acute effects of static stretching. Such studies reported a significant decrease in postural control after static stretching of calf muscles [23,24,35] which could also be related to deterioration of ankle force sense, and may consequently impair balance control with ankle strategy. Reduced ankle force sense acuity can be also explained as being caused by reduced muscle power output and muscle force after static stretching [36,37] thus affecting force sense perception. These changes could be due to mechanical adaptations of muscle-tendon unit and adjustments in the neural system. Static stretching may also cause peripheral changes such as reduced passive MTU stiffness and increased MTU length [24] which affects muscle spindle receptors and Golgi tendon organs. Changes at the neural level are associated with decreased afferent input into the motor neuron pool, and therefore on reduced tonic reflex activity [25]. It seems that the previously mentioned mechanisms underlying static stretching had a significant effect on reduced ankle force acuity. In our case, impaired ankle force acuity is probably not caused by thixotropy because subjects actively moved their ankle through full range of motion to avoid it [28].

Even though greater force sense errors were expected after concentric contractions, our results suggest that this intervention protocol did not have a significant effect. Impaired ankle force sense relative to the control condition was only observed when all effects for dorsiflexion (DF-SUM) were taken into account. Greater AE of DF compared to PF could be due to different function and muscle structure of these two muscle groups, where PF could be less susceptible to fatigue. One study reported that concentric contractions of ankle PF muscles lead to less accurate and less consistent force matching performance [12], but, in this study, subjects performed concentric contractions until maximal exhaustion. Alteration of another aspect of kinaesthesia (joint position sense) has been noted after isometric contractions for DF muscles [38], while some other studies using different fatiguing protocols showed no impaired kinaesthesia of ankle joint [13,18]. We can conclude that concentric contractions of 30% 1RM did not affect PF and DF muscles to cause significant impact on force sense of the ankle. Other factors or differences could be related to the young age of our subjects and their presumably good physical condition since they were healthy sport science students.

Results for the one-legged balance task showed no significant acute effect on ankle force matching performance. While standing on unstable surface information derived from somatosensory system becomes ambiguous [27], in contrast, vestibular [39] and visual [40] information is upweighted. This takes place because changes in the length of muscles in the lower extremity are not coherent with changes in body orientation relative to gravity [41]. Other studies also reported reduced gains of proprioceptive reflexes when balancing on unstable surfaces [42,43], however, we assume that changes in the use of afferent information vanish after such a short period of one-legged balancing. It is important to note that the influence of the balance task is dependent on subjects having previously practiced the task [27] and that the one-legged balance task might impose different difficulty for each subject as a result, leading to contrasting adaptations. Overall, the balance task probably did not induce any greater mechanical or neural adaptations influence on the force sense of the ankle, or they balanced each other out.

In summary, the present findings showed significant deterioration of ankle force sense after the static stretching task, while there was no significant changes after concentric contractions and the balance task. This could be useful information for proper sequence planning during the same exercise unit. While some studies have suggested that static stretching may be effective in injury prevention [44–47] and increasing joint range of motion [48], other studies have pointed out that stretching could increase the risk of injuries [30,49–52]. Despite that, it can also have a negative effect on immediate physical performance in jumping, sprinting, running, or balance [1,50]. The present findings may support this notion further, on activities that include great deal of balance, coordination, and precision, at least for the ankle joint. Finally, it would be interesting to investigate these effects in the elderly who have less accurate postural control and greater risk of falling. The total stretching, balance and concentric contraction times were not matched and may influence on the results of the study. However, it should be noted that these exercise modalities generally have different duration in practice so our results have ecological validity. Although our measurements were undertaken in isometric conditions, which could be a possible limitation of our study, the results clearly highlighted the potential deleterious effects of static stretching on ankle force sense acuity.

## Supporting information

S1 File Individual data of the error scores for force sense test. (XLSX)
